# Elevated Aminopeptidase P Attenuates Cerebral Arterial Responses to Bradykinin in Fawn-Hooded Hypertensive Rats

**DOI:** 10.1371/journal.pone.0145335

**Published:** 2015-12-18

**Authors:** Md Abdul Hye Khan, Amit Sharma, Kevin R. Rarick, Richard J. Roman, David R. Harder, John D. Imig

**Affiliations:** 1 Department of Pharmacology & Toxicology, Medical College of Wisconsin, Milwaukee, Wisconsin, United States of America; 2 Cardiovascular Center, Medical College of Wisconsin, Milwaukee, Wisconsin, United States of America; 3 Department of Pharmacology & Toxicology, University of Mississippi Medical Center, Jackson, Mississippi, United States of America; 4 Department of Physiology, Medical College of Wisconsin, Milwaukee, Wisconsin, United States of America; Fraunhofer Research Institution of Marine Biotechnology, GERMANY

## Abstract

Cerebral arterial myogenic and autoregulatory responses are impaired in Fawn Hooded hypertensive (FHH) rats. Cerebral autoregulatory responses are restored in the congenic rat strain in which a segment of chromosome 1 from the Brown Norway (BN) rat was transferred into the FHH genetic background (FHH.1BN). The impact of this region on cerebral arterial dilator responses remains unknown. Aminopeptidase is a gene that was transferred into the FHH genetic background to generate the FHH.1BN rats and is responsible for degradation of the vasodilator bradykinin. Thus, we hypothesized that FHH rats will have increased aminopeptidase P levels with impaired cerebral arterial responses to bradykinin compared to BN and FHH.1BN rats. We demonstrated higher cerebral arterial expression of aminopeptidase P in FHH compared to BN rats. Accordingly, we demonstrated markedly impaired cerebral arterial dilation to bradykinin in FHH compared to BN rats. Interestingly, aminopeptidase P expression was lower in FHH.1BN compared to FHH rats. Decreased aminopeptidase P levels in FHH.1BN rats were associated with increased cerebral arterial bradykinin-induced dilator responses. Aminopeptidase P inhibition by apstatin improved cerebral arterial bradykinin dilator responses in FHH rats to a level similar to FHH.1BN rats. Unlike bradykinin, cerebral arterial responses to acetylcholine were similar between FHH and FHH.1BN groups. These findings indicate decreased bradykinin bioavailability contributes to impaired cerebral arterial dilation in FHH rats. Overall, these data indicate an important role of aminopeptidase P in the impaired cerebral arterial function in FHH rat.

## Introduction

A number of studies have demonstrated that impaired endothelial function is a strong predictor of cardiovascular events like stroke, myocardial infarction, congestive heart failure, and sudden cardiac death [1–3]. In line with these findings, it is also demonstrated that impaired cerebrovascular endothelial function leads to cerebrovascular disease including stroke [4–5]. In the present study, we investigated vasodilatory function in cerebral arteries of Fawn Hooded hypertensive (FHH) rats, which is a genetic model of mild hypertension and reported to develop cerebral arterial dysfunction associated with impaired autoregulation of cerebral blood flow [6–8]. A previous study compared cerebrovascular autoregulatory function in FHH rats with a genetically modified congenic strain of FHH rat (FHH.1BN). In this congenic FHH.1BN rat a 2.4-Mbp region of RNO1 from 258.8 to 261.2 Mbp on chromosome 1 was transferred from normal Brown Norway (BN) rats into FHH genetic background [9]. It was demonstrated that transfer of this region from BN to FHH.1BN rats restores cerebral vascular autoregulatory responses and reduces cerebral injury after transient occlusion and reperfusion of the middle cerebral arteries [8].

Not known is whether or not, this chromosomal region harboring 16 genes is involved in regulating other aspects of cerebral arterial function such as vasodilation in FHH rats. Indeed, the genetic analysis of the 2.4-Mbp region on the chromosome 1 of the FHH rat demonstrated that the gene for x-prolyl aminopeptidase (aminopeptidase P), a gene involved in bradykinin metabolism is present [10–11]. Bradykinin is a nonapeptide produced locally in different tissues and exhibits potent vasodilatory and cardioprotective effects through its effects on nitric oxide, prostaglandins, and endothelium-derived hyperpolarizing factor [12]. Aminopeptidase P inactivates bradykinin by hydrolyzing the N-terminal Arg^1^-Pro^2^ bond [11,13]. It has been reported that specific inhibition of aminopeptidase P by apstatin increased vasodepressor responses to bradykinin in rats [14]. The contribution of aminopeptidase P in bradykinin metabolism has also been demonstrated in humans [13]. Consequently, in the present study, we hypothesized that compared to BN and FHH.1BN rats the FHH rats will have a higher level of aminopeptidase P that will impair bradykinin-mediated cerebrovascular dilator responsiveness. We further hypothesized that the congenic FHH.1BN rats will have lower aminopeptidase P levels and improved bradykinin-mediated cerebral arterial dilator responses than FHH rats.

## Materials and Methods

### Chemicals

Unless and otherwise mentioned, all chemicals used in this study were purchased from Sigma-Aldrich (St. Louis, MO, USA).

### Animals

**Fig 1:**
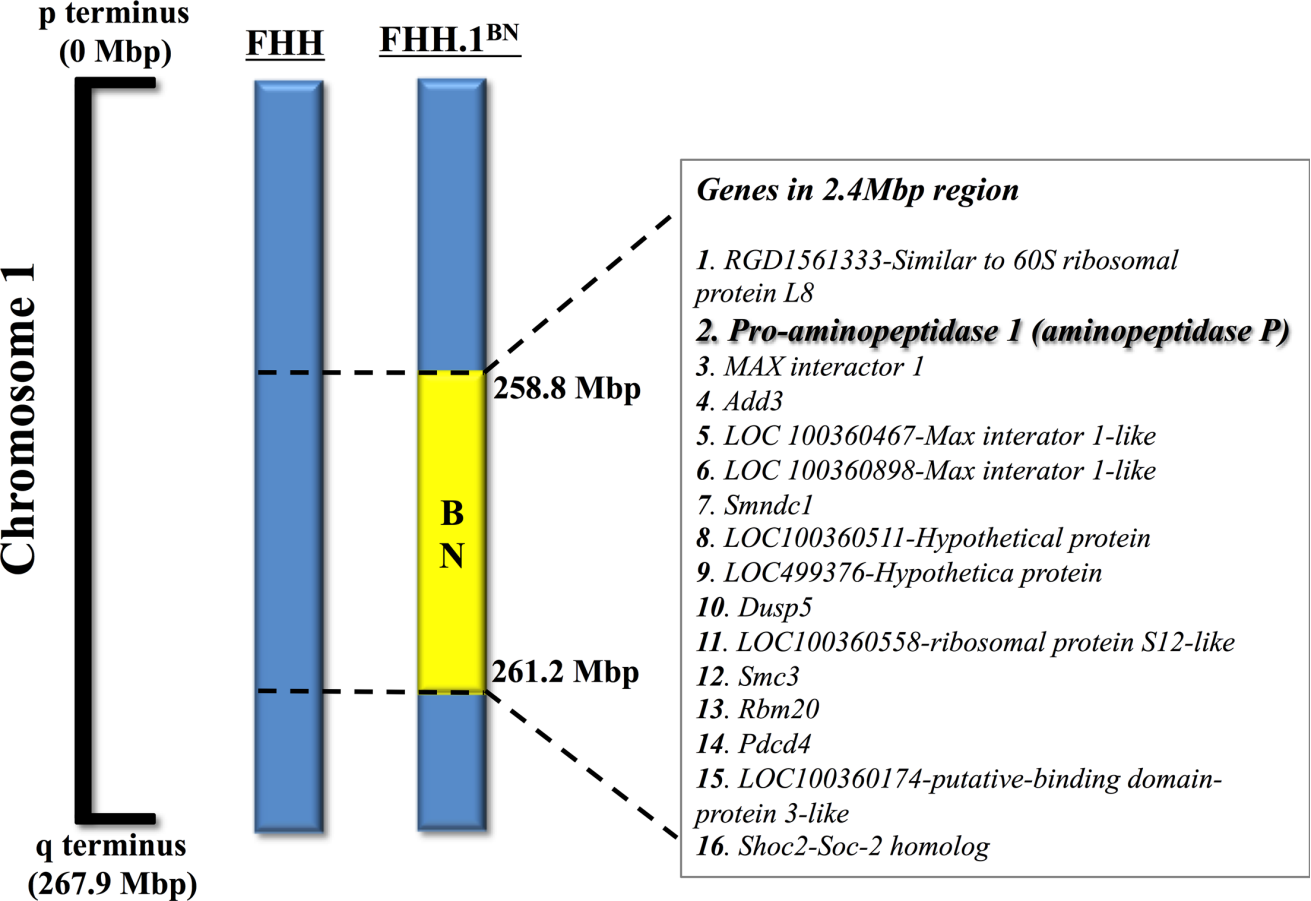
Illustrative depiction of the genetic map of introgressed region in FHH.1BN congenic rats. Genomic segments from the fawn-hooded hypertensive (FHH) and Brown Norway (BN) rat are presented as blue and yellow bars, respectively. Aminopeptidase P and 15 other genes harbored in the 2.4 Mbp region of interest on rat chromosome 1 are shown. The concept of this figure is adapted from earlier studies [8,9].

Experiments were conducted using 9–12 weeks old male Fawn-Hooded rat (FHH), a genetically modified congenic strain of FHH rat (FHH.1BN) and Brown Norway (BN) rats. A genetic map of the introgressed region in chromosome 1 of the FHH.1BN congenic strain is shown in Fig 1, which is adapted from earlier studies [8,9]. Animal protocols were in accordance with National Institutes of Health guidelines and approved by the Institutional Animal Care and Use Committee at the Medical College of Wisconsin. Animals were fed normal chow throughout the experiment and were housed under conditions of constant temperature and humidity with a 12:12h light–dark cycle. Animals were allowed to adapt to these conditions for several days before starting any experimental procedures. The rats were decapitated under anaesthesia (Isoflurane, Piramal Critical Care Inc., Bethlehem, PA, USA) and the cerebral arteries were very quickly collected in oxygenated (95% O_2_/5% CO_2_) Krebs physiological salt solution right before their use.

### Real-Time PCR Analyses

Real-Time PCR analysis was carried out to assess the expression of aminopeptidase P. Total RNA was isolated from middle cerebral artery homogenates using RNeasy Mini Kit (Qiagen, USA) according to the manufacturer’s instructions. The mRNA samples were quantified by spectrophotometry reverse-transcribed to cDNA using iScript™ Select cDNA Synthesis Kit (Bio-Rad, USA). Specific oligonucleotide primer designed and synthesized (Integrated DNA Technologies, Inc., USA) for PCR amplification of aminopeptidase P (forward sequence: 5’-GCC AAG CAG ATG GAC AAC A-3’ and reverse sequence: 5’-TTC TCC TTC ACA GGC ACG A-3’), bradykinin type receptor 1 (forward sequence: 5’-CTG GCC CTT CGG AAC TGA-3’ and reverse sequence: 5’-CAA ACA GGT TGG CCT TGA TGA C-3’), and bradykinin receptor type 2 (forward sequence: 5’-ATC ACC ATC GCC AAT AAC TTC GA-3’ and reverse sequence: 5’-CAC CAC GCG GCA CAG-3’). Gene expression was quantified by iScript One-Step RT-PCR Kit with SYBR green using MyiQ^™^ Single Color Real-Time PCR Detection System (Bio-Rad Laboratories, Hercules, CA, USA). Each sample was run in duplicate, and the comparative cycle threshold (C_t_) method was used to quantify fold increase (2^–ΔΔCt^) in the expression of aminopeptidase P gene.

### Immunoblotting

Twenty micrograms of homogenized middle arterial samples were separated by SDS-PAGE on a 12% Tris-glycine gel, and proteins were transferred electrophoretically to a nitrocellulose membrane. Nonspecific binding sites were blocked by incubating the blots overnight at 4°C in a Tris NaCl buffer (TBS) containing 5% nonfat dry milk and 0.1% Tween 20. The primary antibody used was anti-chicken polyclonal antibody for aminopeptidase P (1:1000) (Abcam, Cambridge, MA). The blot was then washed in TBS-0.1% Tween and incubated with the secondary antibody goat anti-chicken (1:20,000) or goat anti-mouse (for tubulin) (Cell Signaling Technology, Danvers, MA) conjugated to horseradish peroxidase for 1h. Detection was accomplished using enhanced chemiluminescence Western blot analysis, band intensity was measured densitometrically, and the values were normalized tubulin.

### Vascular Reactivity Studies

#### Study 1

In sets of vascular experiments, middle cerebral arteries were excised from groups of FHH, FHH.1BN and BN rats and arterial segments were suspended between two glass cannulas in a pressure myograph system (Danish Myo Technology model 111P, Aarhus, Denmark). The bath was oxygenated in 95% O_2_/5% CO_2_ Krebs physiological salt solution (119.0 mmol L^-1^ NaCl, 25.0 mmol L^-1^ NaHCO_3_, 4.6 mmol L^-1^ KCl, 1.2 mmol L^-1^ KH_2_PO_4_, 1.2 mmol L^-1^ MgSO_4_, 1.8 mmol L^-1^ CaCl_2_, 11.0 mmol L^-1^ glucose) at pH 7.4 and 37°C. Under no-flow conditions, the vessel was pressurized from 10 to 80 mmHg in increments of 10 mmHg every three minutes. The vessel was then pressurized to 80 mmHg for 30 min for equilibration and kept at 80 mmHg for the remainder of the experiment. One vessel segment was used per experiment. Lumen diameter measurements were acquired and logged using the myoview 1.2P user interface. The control lumen diameter was measured as a mean over the last minute of the 30 min equilibration period. After being constricted with U46619, a thromboxane mimetic, the diameter was measured as a mean over the last five minutes of a 15 min period. Following U46619 constriction, vessel diameter responses to graded doses of bradykinin or acetylcholine (10^−9^–10^−5^ M) were assessed. The nitric oxide donor, sodium nitroprusside (100 μM) was added to the bath at the end of the experimental period to ensure the vascular integrity. Relaxation responses were plotted as a percentage of relaxation from the maximum contraction.

#### Study 2

As described in *Study 1*, middle cerebral arteries were excised from groups of FHH, FHH.1BN, or BN rats and similarly prepared to assess vessel diameter responses to graded doses of bradykinin (10^−9^–10^−5^ M). The vessel diameter responses were studied in the absence and presence of apstatin (N-[(2S,3R)-3-amino-2-hydroxy-4-phenyl-butanoyl]-L-prolyl-L-prolyl-L-alaninamide), an inhibitor of aminopeptidase P added in the bath (100 μM) during 30-minute incubation period [15–17]. As described in *Study 1*, dilatory responses to bradykinin were plotted as a percentage of relaxation from the maximum contraction.

### Statistics

All data were expressed as mean ± S.E.M. Statistical significance between groups for gene and protein expression data was determined by one-way analysis of variance followed by post-hoc test using GraphPad Prism^®^ Version 4.0 software (GraphPad Software Inc, La Jolla, CA, USA). The significance of differences in overall responses to bradykinin or acetylcholine was determined with two-way analysis of variance for repeated measures followed by Duncan’s multiple-range post-hoc test (GraphPad Prism^®^ Version 4.0). Probability values of P <0.05 were considered significant.

## Results

### Cerebral Arterial Aminopeptidase P Is Higher in FHH than BN and FHH.1BN Rats

**Fig 2:**
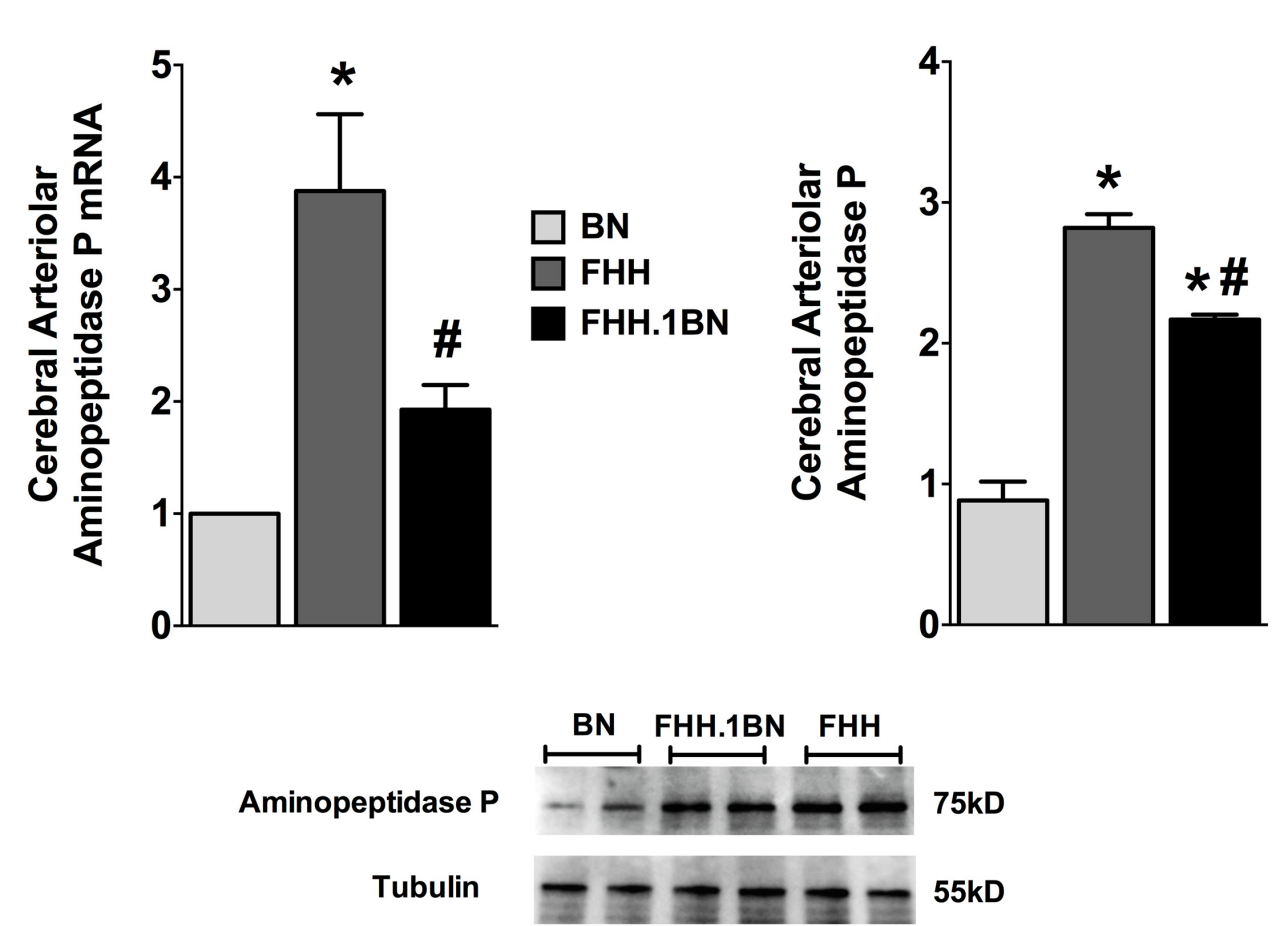
Cerebral arterial aminopeptidase P expression. Cerebral arteriolar aminopeptidase P mRNA and protein expression in different experimental groups. Values are mean± S.E.M., *P< 0.05. vs. BN and #P< 0.05. vs. FHH, n = 6.

In the present study, we determined middle cerebral arterial aminopeptidase P mRNA and protein expression in the different experimental groups. Aminopeptidase P mRNA expression was 4-fold higher in FHH compared to BN rats. Interestingly, in the congenic FHH.1BN rats, which have replacement of a 2.4-Mbp region harboring the aminopeptidase P gene on RNO1 of chromosome 1, we found a 50% lower mRNA aminopeptidase P expression compared to FHH rats (Fig 2). Similar to mRNA expression, we also found a markedly higher level of cerebral arterial aminopeptidase P protein expression in FHH compared to BN rats. Cerebral arterial aminopeptidase P protein expression in the congenic FHH.1BN is 40% lower compared to FHH rats (Fig 2). These data demonstrate increased aminopeptidase P levels in FHH compared to BN and FHH.1BN rats.

### Cerebral Arterial Diameters in BN, FHH, and FHH.1BN Rats

Cerebral arterial diameters were not different between the experimental groups. Diameters averaged 118.0 ± 5.8 μm (n = 24) in BN, 131.8 ± 6.1 μm (n = 28) in FHH, and 130.2 ± 6.5 μm (n = 27) in FHH.1BN rats. U46619 constricted cerebral arterial to a similar level. Cerebral arterial diameters averaged 53.9 ± 5.6 μm (n = 24) in BN, 53.6 ± 4.9 μm (n = 28) in FHH, and 50.9 ± 5.0 μm (n = 27) in FHH.1BN rats following the addition of U46619.

### Impaired FHH Cerebral Arterial Dilation to Bradykinin Are Rescued in FHH.1BN Rats

**Fig 3:**
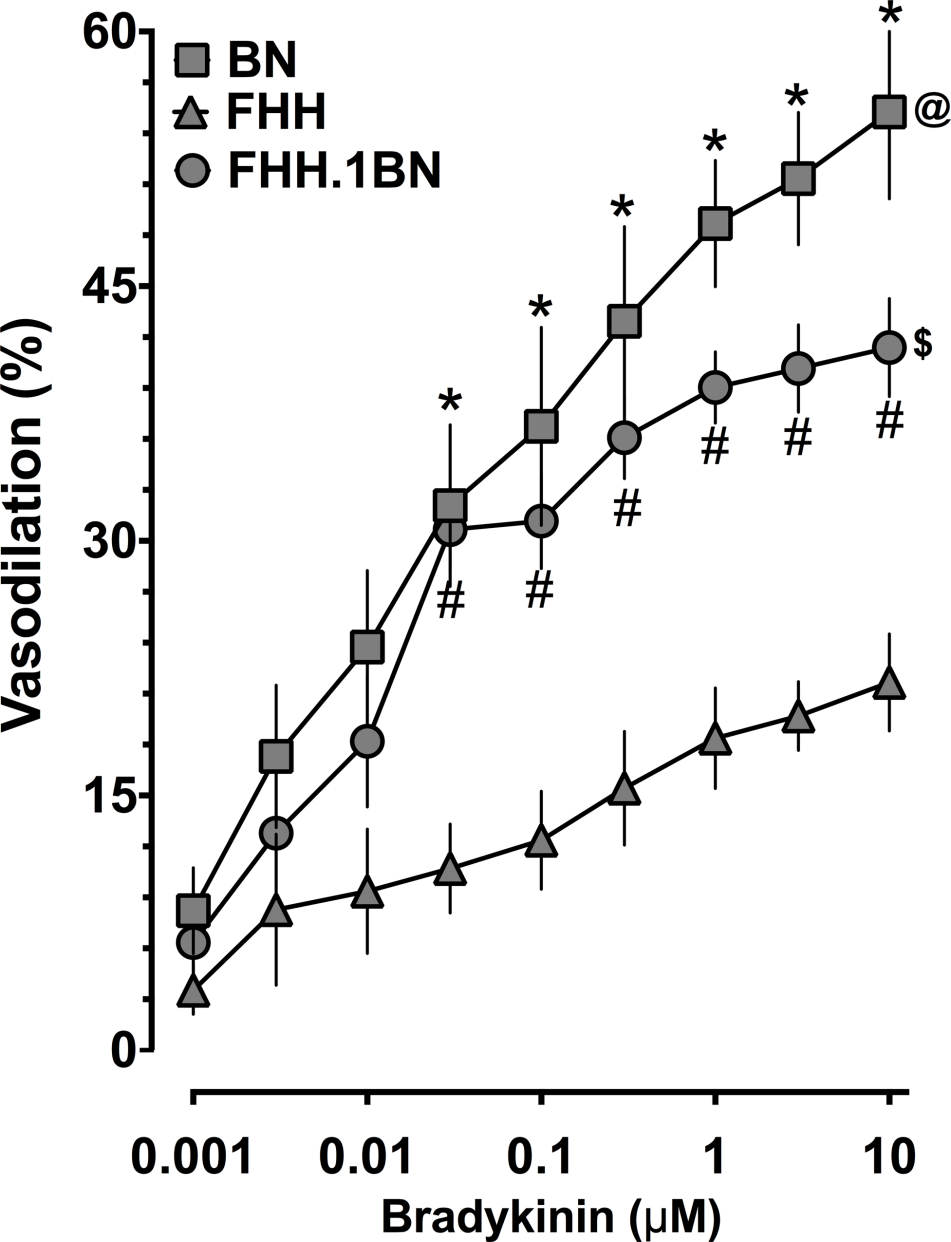
Cerebral arterial responses to bradykinin. Cerebral arterial responses to bradykinin in BN, FHH, and FHH.1BN rats. *Indicates significant difference from the same concentration of bradykinin between BN and FHH. #Indicates significant difference from the same concentration of bradykinin between FHH and FHH.1BN. @ and $ Indicate significant difference in the overall bradykinin response between FHH & BN and FHH & FHH.1BN, respectively. A value of P<0.05 was considered statistically significant. Values are presented as mean± S.E.M., n = 8.

As demonstrated in Fig 3, middle cerebral arterial dilator responses to bradykinin were decreased in FHH compared to BN rats. Replacing chromosome 1 region harboring the aminopeptidase P gene with the BN region (FHH.1BN) resulted in greater cerebral arterial dilator responses to bradykinin than those in FHH rats. Cerebral arterial dilator responses to the nitric oxide donor sodium nitroprusside were similar in all groups and averaged 85.2 ± 3.1% (n = 16) in BN, 79.2 ± 4.3% (n = 20) in FHH, and 79.4 ± 1.8% (n = 19) in FHH.1BN rats. Bradykinin receptor regulation did not contribute to the impaired cerebral arterial responses in FHH or improved responses in FHH.1BN rats. Bradykinin receptor type 1 mRNA expression was 1.6 ± 0.12 (n = 5) in FHH and 0.85 ± 0.07 (n = 5) in FHH.1BN compared to 0.99 ± 0.11 (n = 5) in BN rats. Bradykinin receptor type 2 mRNA expression was 1.4 ± 0.13 (n = 5) in FHH and 0.88 ± 0.05 (n = 5) in FHH.1BN compared to 1.02 ± 0.11 (n = 5) in BN rats. These findings support the notion that the bradykinin-mediated cerebral arterial dilatory function was rescued in congenic FHH.1BN rats.

### Aminopeptidase P Inhibition Improves Cerebral Arterial Dilation to Bradykinin in FHH Rats

**Fig 4:**
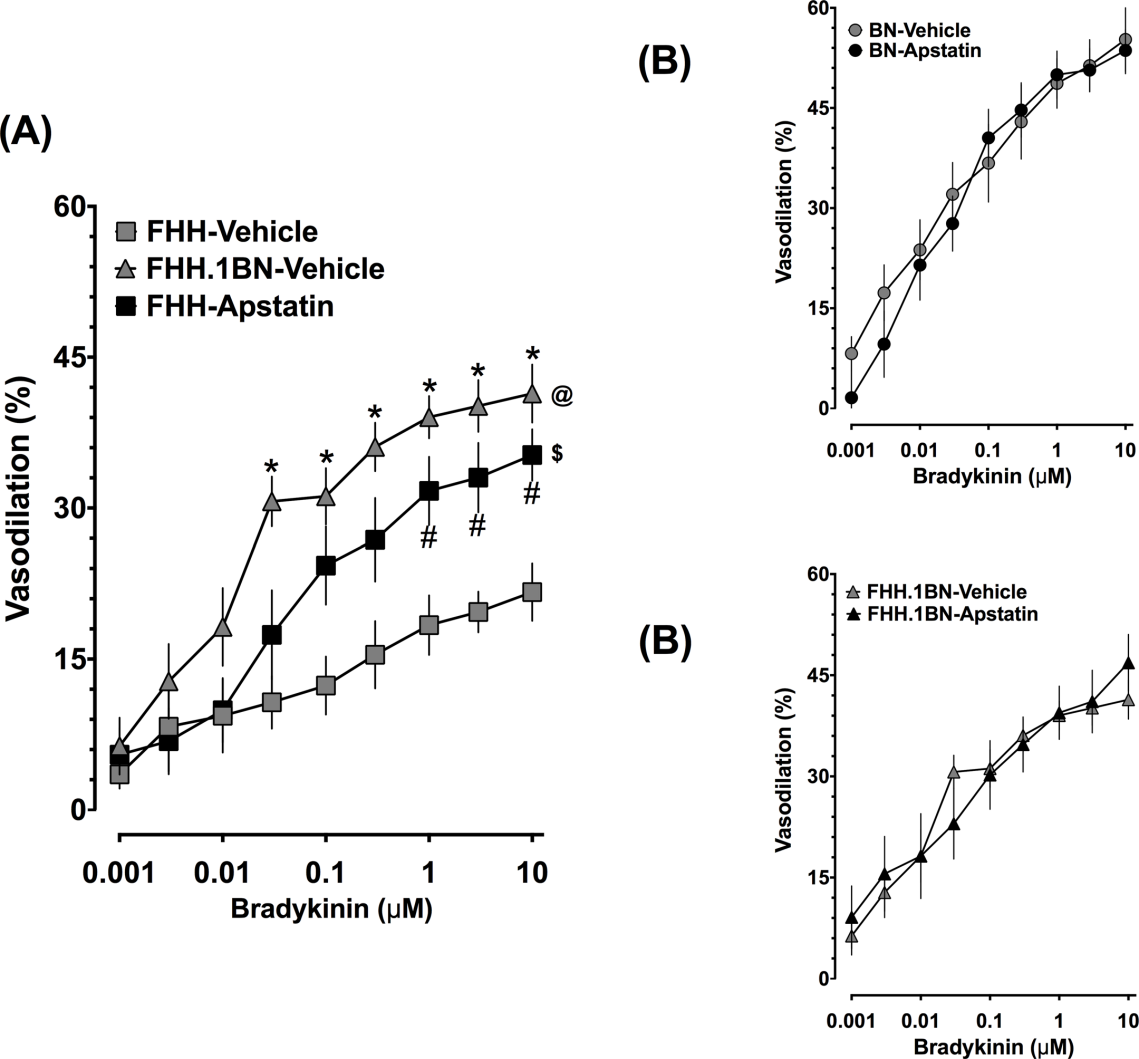
Cerebral arterial responses to bradykinin in the absence and presence of aminopeptidase P inhibition. Cerebral arterial responses to bradykinin in the absence and presence of a specific aminopeptidase P inhibitor, apstatin, in different experimental groups. (A) Apstatin restored cerebral arteriolar responses to bradykinin in FHH rats. *Indicates significant difference from the same concentration of bradykinin between FHH-Vehicle and FHH.1BN-Vehicle. #Indicates significant difference from the same concentration of bradykinin between FHH-Vehicle and FHH-Apstatin. @ and $ Indicate significant difference in the overall bradykinin response in FHH-Vehicle with that in FHH.1BN-Vehicle and FHH-Apstatin, respectively. Cerebral arteriolar bradykinin responses in BN (B) and FHH.1BN (C) rats were not affected by apastatin. A value of P<0.05 was considered statistically significant. Values are presented as mean± S.E.M., n = 8.

In a separate set of experiments we demonstrate that pharmacological aminopeptidase inhibition with apstatin improved bradykinin cerebral arterial dilator responses in FHH rats. In FHH rats, cerebral arterial responses to bradykinin were impaired and apstatin treatment markedly improved cerebral arterial bradykinin responses (Fig 4A). We further demonstrated that apstatin treatment did not alter cerebral arterial responses to bradykinin in BN (Fig 4B) and FHH.1BN (Fig 4C) rats. Additionally, apstatin did not alter the cerebral arterial dilator responses to the nitric oxide donor sodium nitroprusside. Sodium nitroprusside responses were similar in all groups and averaged 82.8 ± 3.8% (n = 8) in BN, 96.3 ± 6.6% (n = 8) in FHH, and 81.4 ± 3.7% (n = 8) in FHH.1BN rats.

### Cerebral Arterial Dilation to Acetylcholine Is Unaltered between FHH and FHH.1BN Rats

**Fig 5:**
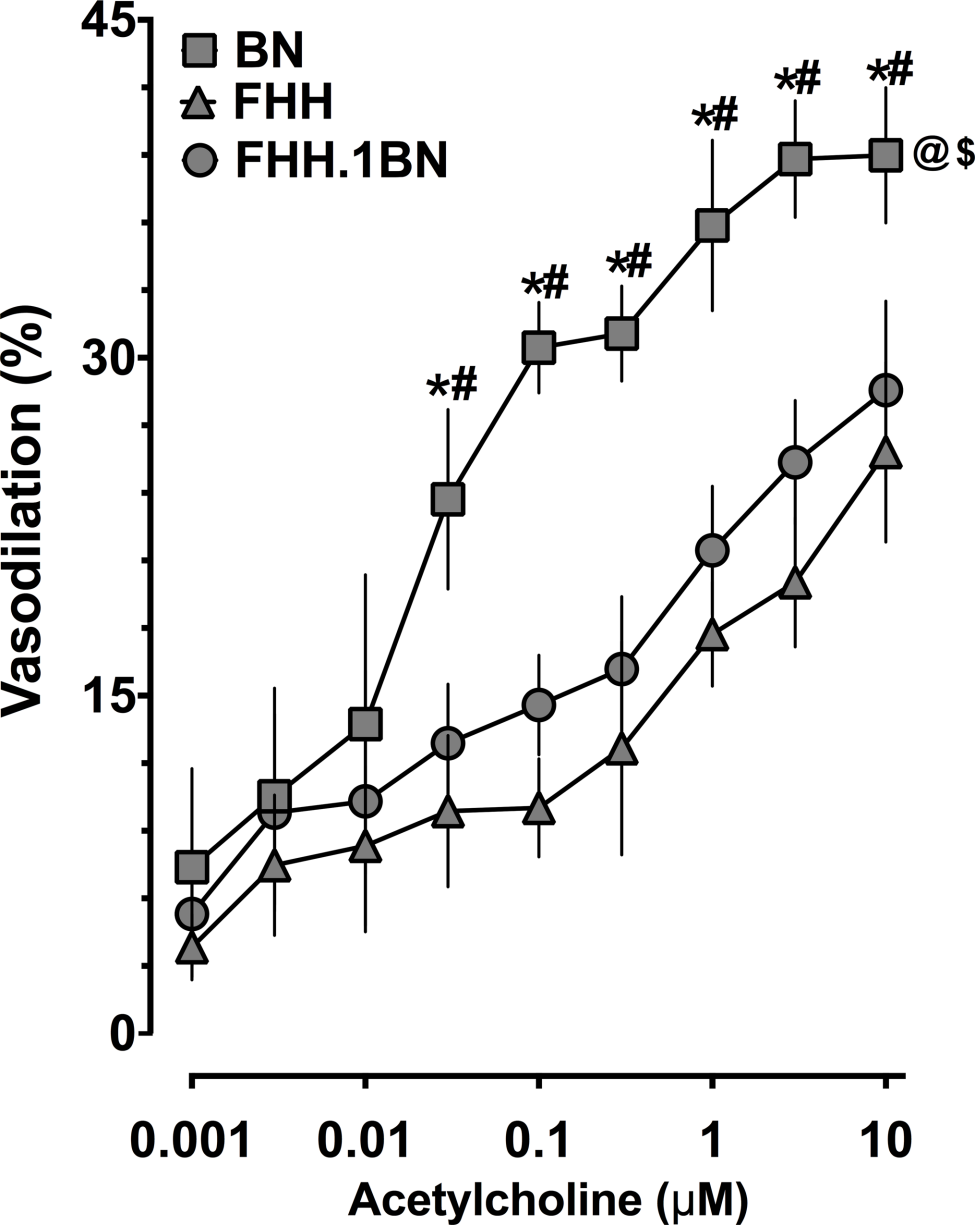
Cerebral arterial responses to acetylcholine. Cerebral arterial responses to acetylcholine in different experimental groups. *Indicates significant difference from the same concentration of acetylcholine between BN and FHH. #Indicates significant difference from the same concentration of acetylcholine between BN and FHH.1BN. @ and $ Indicate significant difference in the overall acetylcholine response in BN with that in FHH and FHH.1BN, respectively. A value of P<0.05 was considered statistically significant. Values are presented as mean± S.E.M., n = 8.

In contrast to our findings of improved bradykinin-mediated cerebral arterial dilator responses in FHH.1BN compared to FHH rats, acetylcholine-mediated cerebral arterial dilator responses in FHH and FHH.1BN were impaired compared to BN rats. Moreover, acetylcholine-induced vasodilator responses in congenic FHH.1BN rats were similar to that in FHH rats (Fig 5). These data demonstrate that cerebral arterial dilator responses to acetylcholine remain impaired in FHH.1BN supporting the notion that replacement of a 2.4-Mbp region of chromosome 1 does not result in non-specific improvement of endothelial-dependent cerebral arterial dilator responses.

## Discussion

In the present study, we demonstrated that FHH rats have higher cerebral arterial aminopeptidase P expression levels along with impaired cerebral arterial dilator responses to bradykinin when compared to BN rats. In the FHH.1BN congenic rats, a segment of 2.4-Mbp region of chromosome 1 (RNO1) was transferred from BN rats to the FHH genetic background. Interestingly, transfer of this chromosomal region in FHH.1BN results in improved renal, systemic, and cerebral blood flow autoregulation, myogenic function, and vascular reactivity [6,8,18,19]. The current findings demonstrate that cerebral arteries of FHH.1BN rats have aminopeptidase P mRNA and protein expression levels that are similar to BN rats. The lower aminopeptidase levels in FHH.1BN rats were associated with improved cerebral arterial dilator responses to bradykinin when compared to FHH rats. These data demonstrate that an increase in aminopeptidase levels in the cerebral arteries of FHH rats impairs cerebral arterial dilator responses to bradykinin.

In regards to the findings of the present study, one can assume a role of hypertension on the impaired cerebral arterial vasodilator response to bradykinin in FHH rats that have been reported to be hypertensive [6,8]. Unfortunately, the term “hypertensive” has been a term used for the fawn-hooded hypertensive (FHH) strain even though this strain exhibits a small 10 mmHg increase in blood pressure relative to other strains between 18 and 21 weeks of age [6,9]. Nevertheless, blood pressure measured by telemetry was not significantly different between FHH and the congenic strain at 9–15 weeks of age and averaged around 120 mmHg [8]. FHH rats utilized in the current study were 9–12 weeks of age that is prior to any elevation in blood pressure. Thus, the phenotypic changes we have observed in the bradykinin cerebral arterial vasodilator responses between FHH and BN rats are not secondary to vascular structural or adaptive changes to hypertension.

Genetic analysis of the 2.4-Mbp (258.8–261.2 Mbp) region of RNO1 of FHH, which is replaced with that of BN harbors 16 genes including ones that are involved vascular autoregulation and myogenic properties of renal and cerebral artery [6,8,9]. Examination of the genes in the region introgressed in the FHH genetic background indicates that there are multiple genes including dual specificity phosphatase 5 (DUSP5), Add3 (adducin), and aminopeptidase P, that could be implicated, in autoregulation, myogenic tone and vasodilator function in FHH rats. Indeed, DUSP5 is a member of the DUSP gene family, which dephosphorylates critical signaling molecules such as MAPK, ERK, and JNK that are involved in pressure- or stretch-induced myogenic responses [20,21]. A recent study demonstrated that cerebral arterial expression of DUSP5 was higher in FHH compared to BN and FHH.1BN rats. In addition, cerebral blood flow autoregulation was improved in DUSP5 knockout and FHH.1BN rats compared to FHH rats that have higher DUSP5 expression [22]. These data support the notion that at least one gene, DUSP5, in this region of chromosome 1 contributed to impaired vascular responses in FHH rats.

Apart from DUSP5, the other two genes present in the 2.4Mbp introgressed region of FHH.1BN rats include Add3 and aminopeptidase P. Add3 is one of the first genes reported to co-segregate with the development of hypertension in a cross of Milan normotensive and Milan hypertensive rats [23]. Add3 promotes the spectrin-actin binding and controls the rate of actin polymerization by capping actin filaments at the plasma membrane [24]. Add3 is also a calmodulin binding protein and serves as a substrate for protein kinase C and Rho kinase, both of which are important regulators of vascular tone [25,26]. Subsequently, evidence has emerged linking mutations in adducin isoforms to the development of hypertension and other forms of cardiovascular disease in rats and humans [27–29].

In the present study, we focused on the aminopeptidase P gene that is present in the transferred RNO1 region in FHH.1BN rats. Aminopeptidase P inactivates bradykinin by cleaving the Arg1-Pro2 bond. Bradykinin is a potent vasodilator peptide that is known to elicit a number of biological responses [14,30,31]. We demonstrated elevated cerebral arterial aminopeptidase P mRNA and protein expression levels in FHH compared to BN and congenic FHH1.BN rats. Accordingly, we also demonstrated that in FHH rats the cerebral arterial dilator responses to bradykinin were markedly impaired compared to BN. Interestingly, FHH.1BN rats had improved cerebral arterial dilator responses to bradykinin that were similar to those observed in BN rats. One cannot totally rule out that the improved cerebral arterial dilator responses to bradykinin in FHH.1BN rats could also be associated with a mechanism other than aminopeptidase P. In this regard, a recent study demonstrated that FHH rats had higher vascular smooth muscle cell K^+^ channel currents compared to BN and FHH.1BN rats [7]. It is possible that the increased K^+^ channel activity in FHH rats hyperpolarizes the cerebral arterials to an extent that they are unable to respond to bradykinin. The fact that FHH.1BN rats did not have a significant improvement in the acetylcholine cerebral arterial dilator response makes this scenario unlikely. Thus, the findings in the current study provide strong evidence for the aminopeptidase P gene in the impaired cerebral arterial dilator responses to bradykinin in FHH rats.

In order to ascertain further the contribution of aminopeptidase P in the impaired cerebral arterial dilator response to bradykinin in in FHH rats, we determined the cerebral arterial response to bradykinin in the presence of apstatin. Apstatin is a specific inhibitor of aminopeptidase P and can block the cleavage of the Arg1-Pro2 bond of bradykinin [15,32]. We demonstrated that in FHH rats, apastatin improved the cerebral arterial dilator response to bradykinin in FHH rats but was without effect on BN or FFH.1BN cerebral arterial bradykinin-mediated dilator responses. These data provide additional evidence for a contribution of increased aminopeptidase P levels in the impaired cerebral arterial dilator response to bradykinin in FHH rats. In accord to these findings, an elevated level of aminopeptidase P has been implicated in the pathophysiology hypertension. Indeed, a role of increased catabolism of bradykinin due to elevated aminopeptidase P has been suggested in oral contraceptive-induced hypertension in women [33]. Thus, aminopeptidase P is a key regulator for bradykinin-mediated cardiovascular actions, and contributes to the pathophysiology of certain type of human hypertension.

In the present study, we also demonstrated that unlike cerebral arterial dilator responses to bradykinin, cerebral arterial dilator responses to another vasodilator, acetylcholine were impaired in FHH rats compared to BN rats but were similar between FHH and FHH.1BN rats. Taken together, the findings of our experimental studies support the notion that the improved bradykinin-mediated cerebral arterial dilator responses in congenic FHH.1BN rats are related to decreased aminopeptidase P and decreased bradykinin degradation. The findings of the present study are important in regards to the role of vasoactive and endothelial-derived factors on cerebral arterial function. Bradykinin is an endogenous vasodilator that is known to contribute to cardiovascular physiology and pathophysiology. An impaired cerebrovascular response to different hormonal and paracrine factors that mediate metabolic regulation of cerebral flood flow has been observed in the FHH rat [8,22]. Further, it has been suggested that abnormalities in the regulation of cerebral blood flow are associated with stroke and can promote cerebral ischemic-reperfusion injury [8,34]. As such, the results obtained in the present study provide information critical to the identification of gene that could be associated with cerebrovascular dysfunction and provide a novel therapeutic target for cerebrovascular diseases.

## References

[1] Widlansky ME, Gokce N, Keaney JF Jr, Vita JA. The clinical implications of endothelial dysfunction. J Am Coll Cardiol. 2003;42: 1149–1160. 14522472 10.1016/s0735-1097(03)00994-x

[2] Halcox JP, Schenke WH, Zalos G, Mincemoyer R, Prasad A, Waclawiw MA et al Prognostic value of coronary vascular endothelial dysfunction. Circulation. 2002;106: 653–658. 12163423 10.1161/01.cir.0000025404.78001.d8

[3] Heitzer T, Schlinzig T, Krohn K, Meinertz T, Munzel T. Endothelial dysfunction, oxidative stress, and risk of cardiovascular events in patients with coronary artery disease. Circulation. 2001;104: 2673–2678. 11723017 10.1161/hc4601.099485

[4] Fagan SC, Hess DC, Hohnadel EJ, Pollock DM, Ergul A. Targets for vascular protection after acute ischemic stroke. Stroke. 2004;35: 2220–2225. 15284446 10.1161/01.STR.0000138023.60272.9e

[5] Rodríguez-Yáñez M, Castellanos M, Blanco M, Mosquera E, Castillo J. (2006) Vascular protection in brain ischemia. Cerebrovasc Dis. 2006;2: 21–29. 10.1159/000091700 16651811

[6] Burke M, Pabbidi M, Fan F, Ge Y, Liu R, Williams JM, et al Genetic basis of the impaired renal myogenic response in FHH rats. Am J Physiol Renal Physiol. 2013;304: F565–F577. 10.1152/ajprenal.00404.2012 23220727 PMC3602705

[7] Pabbidi MR, Mazur O, Fan F, Farley JM, Gebremedhinm D, Harder DR, et al Enhanced large conductance K+ channel (BK) activity contributes to the impaired myogenic response in the cerebral vasculature of Fawn Hooded Hypertensive rats. Am J Physio heart and circulatory physiol. 2014;306(7): H989–H1000. 10.1152/ajpheart.00636.2013 PMC3962634 24464756

[8] Pabbidi MR, Juncos J, Juncos L, Renic M, Tullos HJ, Lazar J, et al Identification of a region of rat chromosome 1 that impairs the myogenic response and autoregulation of cerebral blood flow in fawn-hooded hypertensive rats. Am J Physiol Heart Circ Physiol. 2013;304: H311–H317. 10.1152/ajpheart.00622.2012 23144316 PMC3543673

[9] Williams JM, Burke M, Lazar J, Jacob HJ, Roman RJ. Temporal characterization of the development of renal injury in FHH rats and FHH.1BN congenic strains. Am J Physiol Renal Physiol. 2011;300: F330–F338. 10.1152/ajprenal.00261.2010 21048028 PMC3043997

[10] Ryan JW, Berryer P, Chung AY and Sheffy DH. Characterization of rat pulmonary vascular aminopeptidase P in vivo: Role in the inactivation of bradykinin. J Pharmacol Exp Ther. 1994;269: 941–947. 8014881

[11] Yaron A and Naider F. Proline-dependent structural and biological properties of peptides and proteins. Crit Rev Biochem Mol Biol. 1993;28: 31–81. 8444042 10.3109/10409239309082572

[12] Vanhoutte PM. Endothelium and control of vascular function: State of the art lecture. Hypertension. 1989;13: 658–667. 2661425 10.1161/01.hyp.13.6.658

[13] Kim KS, Kumar S, Simmons WH, Brown NJ. Inhibition of aminopeptidase P potentiates wheal response to bradykinin in angiotensin-converting enzyme inhibitor-treated humans. J Pharmacol Exp Ther. 2000;292: 295–298. 10604961

[14] Kitamura S, Carbini LA, Carretero OA, Simmons WH, Scicli AG. Potentiation by aminopeptidase P of blood pressure response to bradykinin. Br J Pharmacol. 1995;114: 6–7. 7712030 10.1111/j.1476-5381.1995.tb14897.x PMC1510171

[15] Ersahin Cü Simmons WH. Inhibition of both aminopeptidase P and angiotensin-converting enzyme prevents bradykinin degradation in the rat coronary circulation. J Cardiovasc Pharmacol. 1997;30: 96–101. 9268227 10.1097/00005344-199707000-00014

[16] Bagaté K, Develioglu L, Grima M, De Jong W, Simmons WH, Imbs JL, Barthelmebs M. Vascular catabolism of bradykinin in the isolated perfused rat kidney. Eur J Pharmacol. 2000;407: 317–325. 11068029 10.1016/s0014-2999(00)00744-5

[17] Nowak W, Errasti AE, Armesto AR, Santín Velazque NL, Rothlin RP. Endothelial angiotensin-converting enzyme and neutral endopeptidase in isolated human umbilical vein: an effective bradykinin inactivation pathway. Eur J Pharmacol. 2011;667: 271–277. 10.1016/j.ejphar.2011.05.045 21651905

[18] Ochodnický P, Henning RH, Buikema HJ, de Zeeuw D, Provoost AP, Van Dokkum RP. Renal vascular dysfunction precedes the development of renal damage in the hypertensive Fawn- hooded rat. Am J Physiol Renal Physiol. 2010;298: F625–F633. 10.1152/ajprenal.00289.2009 20007352

[19] Lopez B, Ryan RP, Moreno C, Sarkis A, Lazar J, Provoost AP, Jacob HJ, Roman RJ. Identification of a QTL on chromosome 1 for impaired autoregulation of RBF in fawn-hooded hypertensive rats. Am J Physiol Renal Physiol. 2006;290: F1213–F1221. 16303858 10.1152/ajprenal.00335.2005

[20] Tibbles LA, Woodgett JR. The stress-activated protein kinase pathways. Cell Mol Life Sci. 1999;55: 1230–1254. 10487205 10.1007/s000180050369 PMC11147067

[21] Murphy TV, Spurrell BE, Hill MA. Cellular signaling in arteriolar myogenic constriction: involvement of tyrosine phosphorylation pathways. Clin Exp Pharmacol Physiol. 2002;29: 612–619. 12060106 10.1046/j.1440-1681.2002.03698.x

[22] Fan F, Geurts AM, Pabbidi MR, Smith SV, Harder DR, Jacob H, et al Zinc-finger nuclease knockout of dual-specificity protein phosphatase-5 enhances the myogenic response and autoregulation of cerebral blood flow in FHH.1BN rats. PLoS One. 2014;9: e112878 10.1371/journal.pone.0112878 25397684 PMC4232417

[23] Zagato L, Modica R, Florio M, Torielli L, Bihoreau MT, Bianchi G, et al Tripodi G. Genetic mapping of blood pressure quantitative trait loci in Milan hypertensive rats. Hypertension. 2000;36: 734–739. 11082136 10.1161/01.hyp.36.5.734

[24] Matsuoka Y, Hughes CA, Bennett V. Adducin regulation. Definition of the calmodulin-binding domain and sites of phosphorylation by protein kinases A and C. J Biol Chem. 1996;271: 25157–25166. 8810272 10.1074/jbc.271.41.25157

[25] Gardner K, Bennett V. A new erythrocyte membrane-associated protein with calmodulin binding activity. Identification and purification. J Biol Chem. 1986;261: 1339–1348. 3511042

[26] Joshi R, Bennett V. Mapping the domain structure of human erythrocyte adducin. J Biol Chem. 1990;265: 13130–13136. 2376589

[27] Bianchi G, Tripodi G, Casari G, Salardi S, Barber BR, Garcia R, et al Two point mutations within the adducin genes are involved in blood pressure variation. Proc Natl Acad Sci USA. 1994;91: 3999–4003. 8171025 10.1073/pnas.91.9.3999 PMC43710

[28] Tripodi G, Szpirer C, Reina C, Szpirer J, Bianchi G. Polymorphism of gamma-adducin gene in genetic hypertension and mapping of the gene to rat chromosome 1q55. Biochem Biophys Res Commun. 1997;237: 685–689. 9299427 10.1006/bbrc.1997.7173

[29] Efendiev R, Krmar RT, Ogimoto G, Zwiller J, Tripodi G, Katz AI, et al Hypertension-linked mutation in the adducin alpha-subunit leads to higher AP2-mu2 phosphorylation and impaired Na,K_-ATPase trafficking in response to GPCR signals and intracellular sodium. Circ Res. 2004;95: 1100–1108. 15528469 10.1161/01.RES.0000149570.20845.89

[30] Simmons WH, Orawski AT. Membrane-bound aminopeptidase P from bovine lung. Its purification, properties, and degradation of bradykinin. J Biol Chem. 1992;267: 4897–4903. 1537867

[31] Kitamura S, Carbini LA, Simmons WH, Scicli AG. Effects of aminopeptidase P inhibition on kinin-mediated vasodepressor responses. Am J Physiol. 1999;276: H1664–H1671. 10330252 10.1152/ajpheart.1999.276.5.H1664

[32] Prechel MM, Orawski AT, Maggiora LL, Simmons WH. Effect of a new aminopeptidase P inhibitor, apstatin, on bradykinin degradation in the rat lung. J Pharmacol Exp Ther. 1995;275: 1136–1142. 8531074

[33] Cilia La Corte AL, Carter AM, Turner AJ, Grant PJ, Hooper NM. The bradykinin-degrading aminopeptidase P is increased in women taking the oral contraceptive pill. J Renin Angiotensin Aldosterone Syst. 2008;9: 221–225. 10.1177/1470320308096405 19126663

[34] Peterson EC, Wang Z, Britz G. Regulation of cerebral blood flow. Int J Vasc Med. 2011;2011:823525 10.1155/2011/823525 21808738 PMC3144666

